# Effect of left atrial compliance on pulmonary artery pressure: a case report

**DOI:** 10.1186/1476-7120-4-31

**Published:** 2006-08-10

**Authors:** Eloi Marijon, Dinesh Jani, Sébastian Voicu, Phalla Ou

**Affiliations:** 1 Maputo Heart Institute, Mozambique; 2 Department of Pediatric Cardiology, Necker-Enfants Malades Hospital, Paris, France; 3 Department of Pediatric Radiology, Necker-Enfants Malades Hospital, Paris, France

## Abstract

**Background:**

Left ventricular diastolic dysfunction, with secondary atrial pressure elevation, is a well-known concept. On the contrary, effect of left atrial compliance on pulmonary pressure is rarely considered.

**Case presentation:**

We report the echocardiographic case of a 9-year-old child who presented severe rheumatic mitral valve regurgitation with a giant left atrium, in contrast to a normal artery pulmonary pressure, testifying of the high left atrial compliance.

**Conclusion:**

Left atrial compliance is an important determinant of symptoms and pulmonary artery pressure in mitral valve disease.

## Background

Diastolic heart failure, with marked increase in filling pressures secondary to less compliant ventricles is now well evaluated by new echocardiographic modalities. On the other hand, the effect that the left atrial compliance may have on pulmonary artery pressure is not considered in the daily practice. This concept has been studied especially in mitral stenosis, with a wide range of left atrial pressures despite similar mitral valve areas [1,2].

## Case presentation

We report the case of a 9-year-old asymptomatic girl who presented a 3 on 6 holosystolic murmur, discovered during a cross sectional survey estimating rheumatic heart disease prevalence in schoolchildren of Maputo in Mozambique. The apical impulse was displaced to the left, and a third heart sound and a short diastolic rumble reflect the rapid and voluminous left ventricular filling. No hepatomegaly or other right ventricular failure was found. According to the physical examination, the severe mitral regurgitation was estimated on the trans-thoracic echocardiography, using the PISA method (effective regurgitant orifice of 26 mm^2^, body mass index of 0.80 m^2^). Though unfortunately frequent in sub-Saharan Africa, this case draws a particular attention. As seen on the figure, the giant left atrium (73 cm^2^, 2-D four cavities apical view) contrasted with a normal pulmonary artery pressure estimated on Doppler analysis of the minimal tricuspid regurgitation (Doppler velocity 2.45 m/s, giving a systolic pulmonary artery pressure about 29 mmHg), well correlated with normal convexity of the interventricular septum and low speed of the pulmonary diastolic flow. This profile illustrated the important role that left atrial compliance may have on pulmonary artery pressure.

Pressure decay across the mitral valve depends on mitral valve area, trans-mitral gradient, and atrio-ventricular compliance [1]. Atrio-ventricular compliance can be estimated by Doppler echocardiography from the ratio of mitral valve effective orifice area (calculated by the continuity equation, cm^2^) and E-wave downslope (cm/s^2^); according of Flachskampf et al. [3] the net auriculo-ventricular compliance (Cn) can be calculated as follows: Cn = 1270 × (MVA/E dv/dt) (ml/mmHg) [MVA: mitral valve area in cm^2^. E dv/dt: E wave downslope of the mitral Doppler signal in cm/sec^2^]. Atrio-ventricular compliance implies compliance characteristics of the both chambers, atrium and ventricle, as a single unit. When the compliance of either of these chambers is normal, any abnormality of atrio-ventricular compliance reflects abnormal compliance of the other. In case of pure mitral stenosis in young people without additional cardiac pathology, the left ventricle compliance is expected to be normal, and atrio-ventricular compliance is also equivalent to the left atrial compliance, and is a major independent determinant of pulmonary artery pressure measured by catheterization [4]. This low atrial compliance may explain the overestimation of mitral valve area by pressure half-time, in comparison with continuity equation method or planimetry. Moreover, this limitation of the pressure half-time method is well recognized in the setting of percutaneous mitral valvotomy, which induces substantial changes in chamber compliance within a short period of time.

On the opposite, in case of mitral regurgitation, very few data are reported. Echocardiographic atrio-ventricular compliance evaluation may remain difficult because of difficulties to assess to the mitral valve area. In addition, on the contrary of mitral stenosis, in which auriculo-ventricular is essentially dictated by atrial compliance, auriculo-ventricular compliance in mitral regurgitation is more difficult to evaluate. However in mitral regurgitation, atrial size is known to be proportional to its compliance [5], and may explain a normal pulmonary artery pressure in spite of a chronic severe mitral regurgitation.

## Conclusion

In case of severe mitral regurgitation, left atrial size may be a strong predictor of atrial compliance, which is an important determinant of symptoms and pulmonary artery pressure.

## Competing interests

The author(s) declare that they have no competing interests.

## Authors' contributions

EM, DJ carried out the schoolchildren survey to assess to the prevalence of rheumatic heart disease.

EM, DJ did the echocardiography of this case, analyzed the literature, and wrote the paper.

SV, PO have been involved in the final approval of the version to be published, after analyze of the literature.

**Figure 1:**
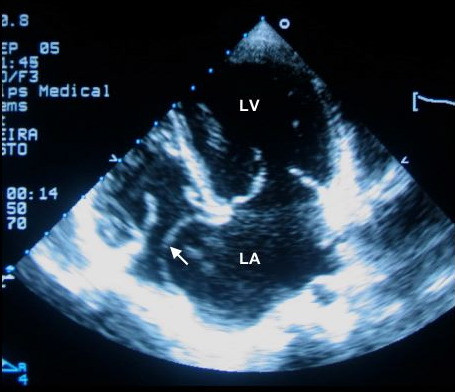
Trans-thoracic echocardiography (2-D apical, four cavities view) showing a giant left atrium (73 cm^2^). Note the marked deviation of the atrial septum (white arrow). LA: left atrium, LV: left ventricle.
